# Growth curve analysis in different generations of Boer x Central Highland goats using alternative estimation models

**DOI:** 10.1371/journal.pone.0293493

**Published:** 2023-11-10

**Authors:** Zeleke Tesema, Alemu Kefale, Belay Deribe, Mekonnen Tilahun, Mesfin Lakew, Getachew Worku Alebachew, Negus Belayneh, Asres Zegeye, Liuel Yizengaw, Kefyalew Alemayehu, Tesfaye Getachew, Damitie Kebede, Mengistie Taye, Solomon Gizaw

**Affiliations:** 1 Debre Birhan Agricultural Research Center, Debre Birhan, Ethiopia; 2 Sirinka Agricultural Research Center, Woldia, Ethiopia; 3 Andasa Livestock Research Center, Bahir Dar, Ethiopia; 4 Amhara Agricultural Research Institute, Bahir Dar, Ethiopia; 5 College of Agriculture and Environmental Sciences, Bahir Dar University, Bahir Dar, Ethiopia; 6 International Center for Agricultural Research in the Dry Areas (ICARDA), Addis Ababa, Ethiopia; 7 International Livestock Research Institute (ILRI), Addis Ababa, Ethiopia; Nasarawa State University, NIGERIA

## Abstract

Growth curve analysis can help to optimize the management, determine nutritional requirements, predict the weight of animals at a specific age, and to select highly productive animals. Therefore, this study aimed to find the best-fitted nonlinear functions to provide a specific shape of the growth curve from birth to yearling age in different generations of Boer x Central Highland goats. Gompertz, Logistic, Brody, Von Bertalanffy, Monomolecular, Negative exponential, and Richards models were evaluated to quantify their ability to describe the biological growth curve. Root mean square error (RMSE), Bayesian information criterion (BIC), adjusted coefficient of determination (AdjR^2^), and Akaike’s information criterion (AIC) were used to evaluate the goodness of fit and flexibility of the models. Data were analyzed using the nonlinear regression procedure of SAS. High AdjR^2^ and lower AIC, BIC, and RMSE values are indicators of best-fitted model. The best-fitting model for the first filial generation (F1), second filial generation (F2), and male goats’ growth data was Brody function, whereas the Richards model, followed by Brody, best described the growth of third filial generation (F3) and female goats. The values of parameter A (asymptotic weight) for F1, F2, F3, female, and male goats based on the Brody model were 30.5±1.32, 28.2±1.38, 24.4±1.04, 27.8±0.94, and 29.8±1.32 kg for F1, F2, F3, female, and male goats, respectively. As per the best-fitted growth function, the asymptotic weight tended to reduce when the filial generation increased. The asymptotic weight for male goats was higher than for female goats. F1 had a slightly small value of parameter K, followed by F2 and F3. Both males and females had similar maturity rates. Based on the Brody function, the correlation between maturation rate and mature weight was high (-0.98, P<0.001). The correlation estimates for A-B and B-K were 0.27 and -0.15, respectively. Brody was best fitted for most goat categories, although Richards, followed by Brody, was best fitted for female and F3 goats. Besides, Brody could be better than Richards due to the ease of interpretation, convergence, and applicability for a small sample size. Therefore, the Brody function can predict the mature body weight, maturation rate, and growth rate of Boer x Central Highland goats and be used to formulate breeding and management strategies for profitable goat farming.

## Introduction

Goats provide both tangible (cash income, meat, milk, and manure for soil fertilizer) and intangible (saving, prestige, insurance, cultural, and ceremonial purposes) benefits to the smallholder farming system [1]. In addition, goats are used as a source of risk mitigation during agricultural failures because of their ability to adapt to harsh climatic conditions. Ethiopia has a huge goat population, with the number of goats estimated to be 50.50 million, with indigenous breeds accounting for 99.97% of the total [2]. One of the most native goat breeds in Ethiopia is the Central Highland goat. Despite their fitness and adaptability, their productivity and economic contribution are below their potential. As a result, many attempts have been made to enhance the productivity of indigenous goats through crossbreeding, within-breed selection, modification of nutrition, and veterinary service. Crossbreeding using Boer goats was one of the attempts. The Boer goat breed is known for its large frame size, high growth rate, and carcass attributes. Thus, indigenous goat breeds in Ethiopia, such as Central Highland goats were crossed with improved Boer goats to improve growth performance and meat production.

Growth is a very important characteristic of living organisms and is defined as an increase in weight and dimension over time [3]. Modeling growth curves provides information for assessing the genetic potential of animals for growth and ascertaining the genetic variability of characteristics linked to growth. In addition, it is used to optimize management, determine the nutritional requirement of animals, and predict the weight of animals at a specific age. Growth curve parameters can also be used as selection and culling criteria in a selective breeding program [4–7]. Thus, modeling the growth curve is important in animal production.

There are various growth models, and due to their sigmoid structure, non-linear growth models are preferable to linear ones [8] and provide the basis for an objective method of estimating growth potential [3]. Nonlinear models (Gompertz, Logistic, Brody, Von Bertalanffy, Monomolecular, Negative exponential, and Richards) are suitable for unbalanced data. Most breeding data are unbalanced, with different numbers of body weight records among levels of factors, as the number of animals decreases over time due to different factors such as death, slaughter, disposal, and other factors. Using conventional methods for such data types leads to bias in the estimated parameters [9]. In addition, non-linear models can describe the weight gain, mature weight, maturing rate, and body weight of animals at any point of the growth trajectory.

Modeling the growth curve was attempted for a few goat breeds or populations using non-linear growth models such as Gompertz, Logistic, Brody, Von Bertalanffy, Monomolecular, Negative exponential, and Richards [4, 7, 10–16]. Nonetheless, breed, flock size, clusters, management level, physical environment, and selection attempts influence growth curves [17]. Besides, in the literature, no studies have been found that describe the growth curve of Boer x Central Highland goats. Therefore, this study aimed to find the best-fitted non-linear functions to provide a specific shape of the growth curve from birth to yearling age in different generations of Boer x Central Highland goats.

## Material and methods

### Ethics statement

Prior to the study, data collection formats and procedures were reviewed and approved by the Researcher of Amhara Regional Agricultural Research Institute, Ethiopia (number Ls/Ru-4/Sr-2015/19) in the annual review forum. Besides, this study was based on data collected from live goats managed at Sirinka Agricultural Research Station without any invasive procedure through close monitoring of researchers. Anesthesia, euthanasia, or animal sacrifice was not part of the study.

### Animals and age-body weight data

The data were obtained from Sirinka Agricultural Research Center sheep and goat breeding station in northeastern Ethiopia. The breeding station is located at an altitude of 1850 m.a.s.l and 11°45’ 00" N and 39°36’ 36" E. The area receives about 950 mm of annual rainfall on average. The area has a moderately warm climate, with average daily temperatures ranging from 13.7 to 26.4°C. Goats were managed semi-intensively, i.e., allowed to graze for about six hours per day on a natural pasture and supplemented with 0.10–0.40 kg of concentrate mixture consisting of wheat bran, Noug seed cake, and salt, based on their physiology, sex, and age. They were housed in semi-open concrete barns at night based on age, physiology, and sex [18].

The data set used in this study comprised 5312 body weight-age records (2963 records on female kids and 2349 records on male kids; 2829 records on F1, 1636 records on F2, and 847 records on F3 kids) from 875 kids that were collected from 2009 to 2018 in Sirinka shoat breeding station, located in Sirinka, northeastern Ethiopia. The numbers of first filial generation (F1), second filial generation (F2), and third filial generation (F3) kids were 434, 293, and 148, respectively. The offspring produced due to a cross between two individuals is called the filial generation. The weight of kids from birth to yearling age was considered in the present study. Kid’s body weight was measured monthly up to six months of age and in three-month intervals afterward.

### Statistical analysis

Levenberg-Marquardt’s iterative approach was used to determine non-linear growth curve model parameters using the NLIN procedure of SAS [19]. The kids’ weights were fitted using seven non-linear models: the Gompertz, Logistic, Brody, Von Bertalanffy, Monomolecular, Negative exponential, and Richards models. Each model was fitted to body weight records for males, females, F1, F2, and F3 kids. The mathematical forms of non-linear growth functions used to describe the growth curves of Boer x Central Highland goats are shown in Table 1.

**Table 1:** Parameters analyzed for models applied in the Boer x Central Highland goats.

Model	NP	Equation	Reference
Gompertz	3	W(t) = Ae^-be-kt^ + ε	[20]
Logistic	3	W(t) = A/(1+be^-kt^) + ε	[21]
Brody	3	W(t) = A(1-be^-kt^) + ε	[22]
Von Bertalanffy	3	W(t) = A(1-be^-kt^)^3^ + ε	[23]
Monomolecular	3	W(t) = A/(1+e^b-kt^) + ε	[24]
Negative exponential	2	W(t) = A(1-e^-kt^) + ε	[25]
Richards	4	W(t) = A(1-be^-kt^)^m^ + ε	[26]

NP, number of parameters; W(t), body weight at age t (month); A, asymptotic or mature weight; and b, an integration constant related to initial animal weight. The value of b is defined by the initial values for W and t, or it is the proportion of mature weight gained after birth; k is the maturation rate, which is interpreted as weight change in relation to mature weight to indicate how fast the animal approaches adult weight; m: inflection point of the curve.

The non-linear models were examined for the goodness of fit using the Bayesian information criterion (BIC), Akaike’s information criterion (AIC), adjusted coefficient of determination (AdjR^2^), and root mean square error (RMSE).

*BIC* was calculated using the following formula:

$$BIC=nln\left(\frac{RSS}{n}\right)+pln(n)$$ (1)

Where *n* is the number of observations (data points), *RSS* is the residual sum of squares, and *p* is the number of parameters in the equation. Smaller values of *BIC* suggest a better fit when comparing the models.

*AIC* was calculated using the following formula:

AIC=n×ln(RSS)+2p (2)

Where *n* is the number of observations (data points), *RSS* is the residual sum of squares, and *p* is the number of parameters in the equation. Smaller values of *AIC* suggest a better fit when comparing the models.

*RMSE* was calculated as follows:

$$RMSE=\sqrt{\frac{RSS}{n-p-1}}$$ (3)

Where *n* is the number of observations (data points), *RSS* is the residual sum of squares, and *p* is the number of parameters in the equation. Smaller values of *RMSE* suggest a better fit when comparing the models.

The adjusted coefficient of determination (*AdjR*^*2*^) was computed as follows:

$$Adjusted\;R^{2}=1-\frac{RSS/dfe}{TSS/dft}$$ (4)

Where *TSS* is the total sum of squares, *dft* is the degrees of freedom *n– 1* of the estimate of the population variance of the dependent variable, and *dfe* is the degrees of freedom *n–p– 1* of the estimate of the underlying population error variance. *The model sum of squares plus the error sum of squares equals the total sum of squares*. A higher value of the adjusted coefficient of determination indicates the best-fit model. The difference between body weight of males and females, and also between the actual and predicted body weight based on selected models was evaluated using a T-test.

## Results and discussion

### Body weight at different ages

The mean (kg), standard deviation, minimum, maximum, and coefficient of variation of body weight from birth to yearling age are presented in Table 2. The range between minimum and maximum body weight at different ages indicates the presence of variation. The coefficient of variation, which depicts the degree of data variability in a sample with respect to the population mean, is a helpful statistic for comparing the degree of variation between data series. The coefficient of variation for the body weight of kids from birth to 12 months, which varied from 21.9 to 34.5%, also confirms the presence of variation. According to Owens et al. [27], growth is measured as an increase in mass and includes cell multiplication, cell enlargement, and incorporation of specific components from the environment. Although many variables affect an animal’s growth, they may be categorized into three basic groups: the animal’s gene pool, the nutrients that it receives, and its habitat [28]. Among these factors, the influence of the most important environmental factors on the body weight of Boer crossbred goats was well discussed in the study of Tesema et al. [18]. In this study, males had significantly higher body weight compared to their female counterparts.

**Table 2:** Descriptive statistics for body weight of Boer x Central Highland goats.

Age	Sex	N	Mean	SD	SE	Minimum	Maximum	CV (%)	T-value	P-value
BWT	F	468	2.47	0.55	0.03	1.00	4.20	22.9	-2.79	0.0054
	M	407	2.58	0.61	0.03	1.00	4.20			
1 month	F	368	4.90	1.26	0.07	2.53	9.13	26.8	-3.32	0.0009
	M	304	5.24	1.44	0.08	2.27	9.73			
2 month	F	368	7.25	2.22	0.12	3.27	14.40	31.7	-3.23	0.0013
	M	304	7.85	2.54	0.15	3.33	15.47			
3 month	F	368	9.61	3.21	0.17	4.00	20.00	34.5	-3.17	0.0016
	M	304	10.45	3.67	0.21	4.00	21.20			
4 month	F	303	9.96	2.26	0.13	5.27	17.27	23.5	-2.28	0.0232
	M	233	10.43	2.51	0.16	5.47	17.33			
5 month	F	303	11.80	2.79	0.16	6.13	20.63	24.4	-2.24	0.0258
	M	233	12.37	3.08	0.20	6.23	20.67			
6 month	F	303	13.64	3.31	0.19	7.00	24.00	25.0	-2.20	0.0279
	M	233	14.31	3.66	0.24	7.00	24.00			
9 month	F	262	17.36	3.85	0.24	9.00	27.00	22.6	-2.67	0.0079
	M	190	18.37	4.21	0.31	9.00	27.40			
12 month	F	218	19.80	4.43	0.30	12.00	32.00	25.3	-2.58	0.0102
	M	133	21.26	6.08	0.53	12.00	35.20			

BWT, birth weight; F, female; M, male; N, number of observation

### Comparison of non-linear growth models

Selecting a model with an inadequate fit can result in growth rates, inflection points, and upper asymptote values with no biological significance [29]. Consequently, picking a suitable growth model is crucial for comprehending animal growth. The results of model comparison for the growth curve of F1, F2, F3, male and female goats under the seven tested non-linear models considering the goodness of fit measures of AIC, BIC, RMSE, and adjR^2^ are shown in Tables 3 and 4. Among the evaluated models, Richards was not converged for F1, F2, and male goats. The Brody followed by Von-Bertalanffy model provided the lowest AIC, BIC, and RMSE values, and these functions had the highest adjR^2^ value compared to other models for F1, F2, and male goats. Based on AIC and BIC values, Richards, followed by Brody, was selected for F3 and female goats. However, these models had almost a similar adjR^2^ and RMSE. Therefore, Brody showed the best fit in F1, F2, and male Boer x Central Highland goats. Likewise, Brody and Richards models best described the growth in F3 and female Boer x Central Highland goats. On the other hand, the Negative exponential function supplied the worst fit of growth in Boer x Central Highland goats due to the highest AIC, BIC, and RMSE values and lowest adjusted R^2^ value. Indeed, the Brody function could be easy to interpret compared with Richards, which had four parameters. Besides, it is not easy to obtain convergence in the Richards function [16, 30] and as observed in this study. In addition, according to Brunner and Kühleitner [31], the Brody model is appropriate for a small sample size and for a sample that merges both male and female animals. Hence, the advantage of the Brody model is more pronounced in developing countries, which havea small sample size for growth curve analysis. Therefore, using Brody model would be better than Richards due to the ease of interpretation, convergence, and applicability for a small sample size.

**Table 3:** Estimated growth curve parameters for different generations of Boer x Central Highland goats from different non-linear models.

Genotype	Growth curve parameters				Goodness of fit test			
	A	B	K	m	BIC	AIC	RMSE	AdjR^2^
**F1**								
Gompertz	22.6±0.40	1.91±0.02	0.22±0.007		8493	69651	2.89	0.937
Logistic	20.8±0.26	4.54±0.11	0.37±0.009		8673	69830	2.92	0.935
Brody	30.5±1.32	0.91±0.003	0.08±0.006		8339	69496	2.85	0.938
Von Bertalanffy	23.9±0.52	0.49±0.004	0.18±0.006		8434	69591	2.87	0.937
Monomolecular	20.8±0.26	1.51±0.02	0.37±0.009		8673	69830	2.92	0.935
Negative exp.	22.4±0.39	-	0.16±0.005		9597	70755	3.12	0.926
Richards	-	-	-	-	Not converged			
**F2**								
Gompertz	21.8±0.48	1.89±0.03	0.25±0.01		5424	66582	3.04	0.926
Logistic	20.4±0.31	4.48±0.16	0.40±0.01		5644	66801	3.09	0.924
Brody	28.2±1.38	0.90±0.005	0.10±0.008		5239	66396	3.00	0.928
Von Bertalanffy	22.9±0.60	0.48±0.006	0.20±0.009		5352	66510	3.03	0.927
Monomolecular	20.4±0.33	1.50±0.03	0.40±0.01		5644	66801	3.09	0.924
Negative exp.	21.7±0.49	-	0.18±0.007		6460	67618	3.28	0.915
Richards	-	-	-	-	Not converged			
**F3**								
Gompertz	20.7±0.48	1.89±0.05	0.30±0.01		428	61585	3.13	0.939
Logistic	19.7±0.36	4.45±0.23	0.45±0.02		681	61839	3.00	0.936
Brody	24.4±1.04	0.89±0.008	0.13±0.01		232	61389	2.91	0.940
Von Bertalanffy	21.4±0.57	0.48±0.09	0.24±0.01		347	61504	2.93	0.939
Monomolecular	19.7±0.36	1.49±0.05	0.45±0.02		681	61839	3.00	0.936
Negative exp.	21.0±0.54	-	0.21±0.01		1291	62448	3.14	0.931
Richards	27.7±4.18	0.95±0.03	0.08±0.003	0.77±0.13	218	61376	2.90	0.940

A, asymptotic weight or mature weight; and b, an integration constant related to initial animal weight. The value of b is defined by the initial values for W and t; k, the maturation rate, which is interpreted as weight change in relation to mature weight to indicate how fast the animal approaches adult weight; m, inflection point of the curve

**Table 4:** Estimated growth curve parameters of male and female Boer x Central Highland goats from different non-linear models.

Sex	Growth curve parameters				Goodness of fit test			
	A	B	K	m	BIC	AIC	RMSE	AdjR^2^
**Female**								
Gompertz	21.5±0.32	1.91±0.02	0.24±0.007	-	8236	69393	2.77	0.940
Logistic	19.9±0.22	4.53±0.11	0.39±0.009		8440	69598	2.81	0.938
Brody	27.8±0.94	0.90±0.003	0.09±0.005		8074	69232	2.74	0.942
Von Bertalanffy	22.6±0.40	0.49±0.004	0.19±0.006		8171	69328	2.75	0.941
Monomolecular	19.9±0.22	1.51±0.02	0.39±0.009		8440	69598	2.81	0.938
Negative exp.	21.6±0.34	-	0.18±0.005		9315	70472	2.99	0.930
Richards	39.2±9.54	0.98±0.01	0.04±0.02	0.74±0.07	8058	69215	2.73	0.942
**Male**								
Gompertz	22.8±0.45	1.90±0.03	0.24±0.009		8373	69531	3.14	0.927
Logistic	21.2±0.31	4.48±0.13	0.39±0.01		8587	69744	3.19	0.925
Brody	29.8±1.32	0.90±0.004	0.09±0.004		8178	69336	3.10	0.929
Von Bertalanffy	24.2±0.56	0.48±0.005	0.19±0.008		8300	69458	3.12	0.928
Monomolecular	21.2±0.31	1.50±0.02	0.39±0.01		8587	69744	3.19	0.925
Negative exp.	22.5±0.44	-	0.18±0.006		9388	70545	3.38	0.916
Richards	-	-	-	-	Not converged			

A, asymptotic or mature weight; b, an integration constant related to initial animal weight. The value of b is defined by the initial values for W and t; k, the maturation rate, which is interpreted as weight change in relation to mature weight to indicate how fast the animal approaches adult weight; m: inflection point of the curve

In line with the current finding, Magotra et al. [15] reported the Brody function as the best model while comparing various growth models in the Beetal goat breed. Waiz et al. [12] estimated the growth curve of Sirohi goats by using five non-linear growth models viz., Brody, Logistic, Gompertz, Weibull, and Richards, and selected Brody as a suitable model. Waheed et al. [4] noted that Brody and Gompertz provided the best fit of growth curve estimates in Beetal goats. Likewise, Freitas [32] reported that Brody, Von-Bertalanffy, and Logistic models were more versatile to fit the growth curve in sheep. Ghavi Hossein-Zadeh [33] evaluated six non-linear functions (Brody, Logistic, Richards, Negative exponential, Bertalanffy, and Gompertz) and found that the Richards model best described growth in male and female Shall sheep. However, Raji et al. [11] compared five different growth models (Brody, Gompertz, Monomolecular, Richards, and Weibull) and found that Gompertz and Monomolecular functions were best for estimating the growth curve in Nigerian goats. On the other hand, Wiradarya et al. [13] and Abdelsattar et al. [14] reported that Gompertz growth curve was the best-fitted model for body weight in Kacang and Laiwu black goats, respectively. The variation of models across studies might be due to the sample size, data structure, appraisal of different types of animals, such as males and females, and the genetic potential of the breeds investigated.

### Growth curve parameter estimates

The goal of crossing indigenous goats with Boer goats is to improve growth performance and meat production. As a result, understanding the biology of body weight and the relationship between age and body weight is crucial for the success of a genetic improvement program. The estimates of growth curve parameters under various models in F1, F2, F3, male, and female Boer x Central Highland goats are presented in Tables 3 and 4. The growth model parameter A represents an asymptotic weight estimate, or the mature weight of animals [3]. Parameter A was the largest for Brody function in F1, F2, and male kids. However, it was high for Richards function in F3 and female kids. Rashad et al. [16] also observed the largest value of parameter A. On the other hand, parameter A was lowest for Logistic and Monomolecular in both sexes and all filial generations. Based on the Brody model, parameter A tended to reduce when the filial generation increased, i.e., F1 had a higher value than F2, and F2 had a higher value than F3. This result concurs with Gaddour et al. [10], who noted that the mature weight and inflected weights of the F1 crossbred kids were higher than F2 genotypes. The heterosis effect could explain the higher performance of F1 in the present study. According to the Brody model, the estimated value of parameter A for male goats was higher than for female goats. This result is in accordance with the reports of Waiz et al. [12] for Sirohi goats and Kheirabadi and Rashidi [34] for Markhoz goats. The hormonal and physiological differences between males and females could be the reason for the superiority of males.

The value of parameter A based on the Brody model in this study was 30.5±1.32, 28.2±1.38, 24.4±1.04, 27.8±0.94, and 29.8±1.32 kg for F1, F2, F3, female, and male goats, respectively. These values are higher than the value (17.97) reported for Raeini Cashmere goats using Gompertz model [7] and the value (8.40 for males and 6.42 for females) noted for the nondescript goat breed [11]. The current result is comparable with the report of Waheed et al. [4] for Beetal goats and Oliveira [35] for Anglo-Nubian goats from the Brody model (29.63). However, Malhado et al. [36] reported a higher value of parameter A for Anglo-Nubian goats (42.96 for the Richards, 42.58 for Brody, and 37.45 kg for the von Bertalanffy models).

As per the Brody function, the ratio of weight gained from birth weight to mature weight in this study varied from 0.89 to 0.91, which is comparable with the value (0.91) reported by Magotra et al. [15] and lower than the result (0.98) noted for the Beetal goats using Brody model [4]. The estimates of parameter B that indicate the proportion of the asymptotic mature weight gained after birth decreased when filial generation increased and was similar for both sexes. However, Magotra et al. [15] noted a higher estimate for males than female Beetal goats than the result in this study.

Parameter K indicates the speed of animal growth to reach mature weight. A small estimated value of parameter K indicates that the animal is late maturing, whereas large values specify late maturation [12, 37]. The estimated value of parameter K differed for the best-fitted growth function and varied from 0.08 for F1 to 0.13 for F3 goats. The estimates for parameter K from a best-fitted model in this study were higher than the result noted for Raeini Cashmere goats (0.017) by Ghiasia et al. [7] and lower than the estimate reported for Sirohi goats [12]. The estimate from Brody was in line with the values reported by Waheed et al. [4] for Beetal goats and close to the estimate (0.13) reported by Magotra et al. [15] for Beetal goats. The variability of estimates is expected, as the estimated value of parameter K is determined by the time unit of age, type, and function of animals.

The value of parameter K tends to increase with the filial generation, and F1 has a slightly small value, followed by F2 and F3 as per the best-fitted growth function. This trend indicates that F3 goats arrive at asymptotic weight earlier than those with lower values of this parameter (F1 and F2). Both males and females had similar values of parameter K, which indicates the absence of a difference in maturation rate among male and female goats. On the contrary, Waiz et al. [12] and Kheirabadi and Rashidi [34] noted that females achieved mature weight earlier than male kids. The aim of crossing indigenous goats with Boer goats was to improve growth and meat production; thus, goats with high asymptotic weight and early maturity could be preferred and may have numerous advantages.

The mature weight and maturation rate are crucial in determining the appropriate slaughtering age for maximum muscle deposition and minimal fat, which might meet customer demands [38]. In this study, the correlation between maturation rate and mature weight was high (-0.98) and significant (P<0.001). This result aligns with the previous studies [5, 34, 39]. The association between asymptotic weight and maturation rate indicates that the early-mature crossbred goats are less likely to exhibit high mature weight. Therefore, selection only for increased mature weight will reduce the maturing rate. The correlation estimates for parameters A-B and B-K based on the best-fitted growth function were 0.27 and -0.15, respectively. A positive phenotypic correlation between A and B indicated that heavy kids at birth had greater weight at maturity, and any increase in the initial body weight of kids could be associated with an increase in mature weight. A positive correlation of A with B is in line with the report of Mohammadi et al. [40] for Kordi sheep. However, as the correlation estimate indicates, heavy kids at birth may not have a high maturation rate. Based on the goal of the breeding program, selecting desirable growth curve parameters can result in an optimal growth curve. Besides, modeling animal growth curves can improve the management and productivity of goats. Thus, goat producers can utilize growth curve parameters to design the ideal feeding schedule for their goats and determine the slaughtering age.

### Actual and predicted body weight

**Fig 1:**
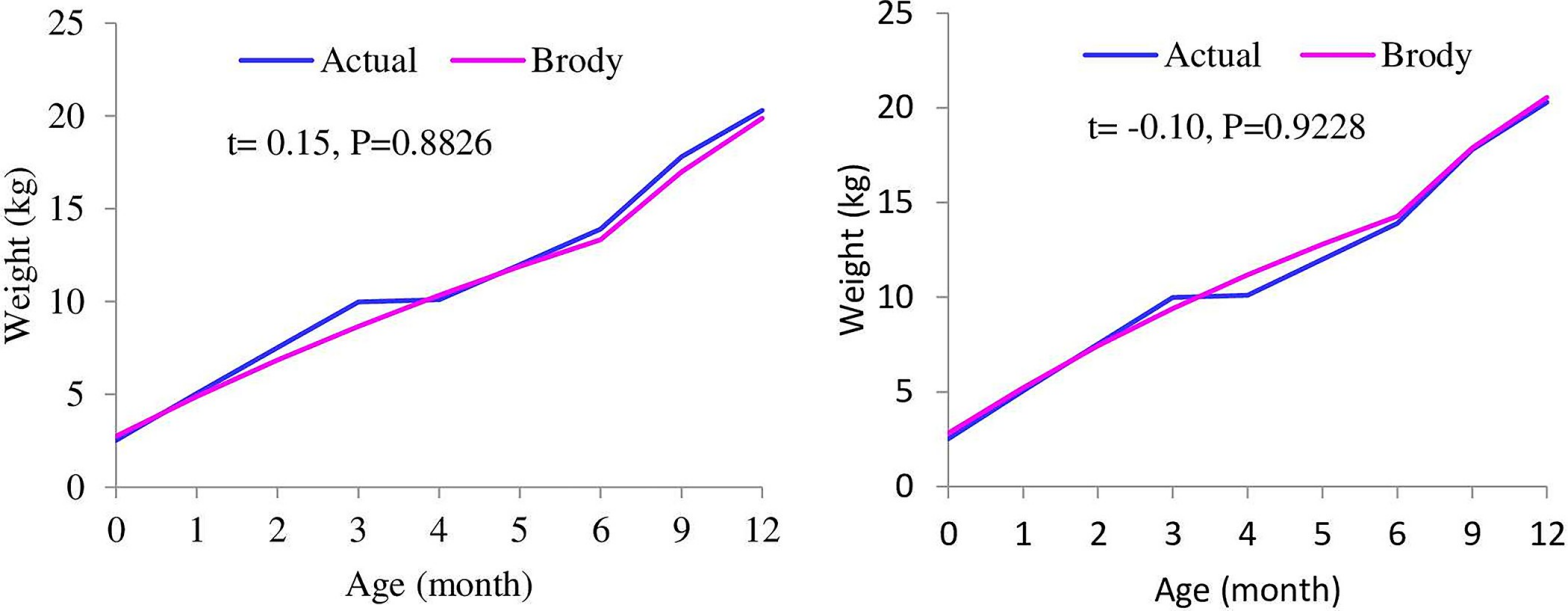
Actual and predicted body weights as a function of age obtained with a best-fitted model for F1 (left) and F2 (right) Boer x Central Highland goats.

**Fig 2:**
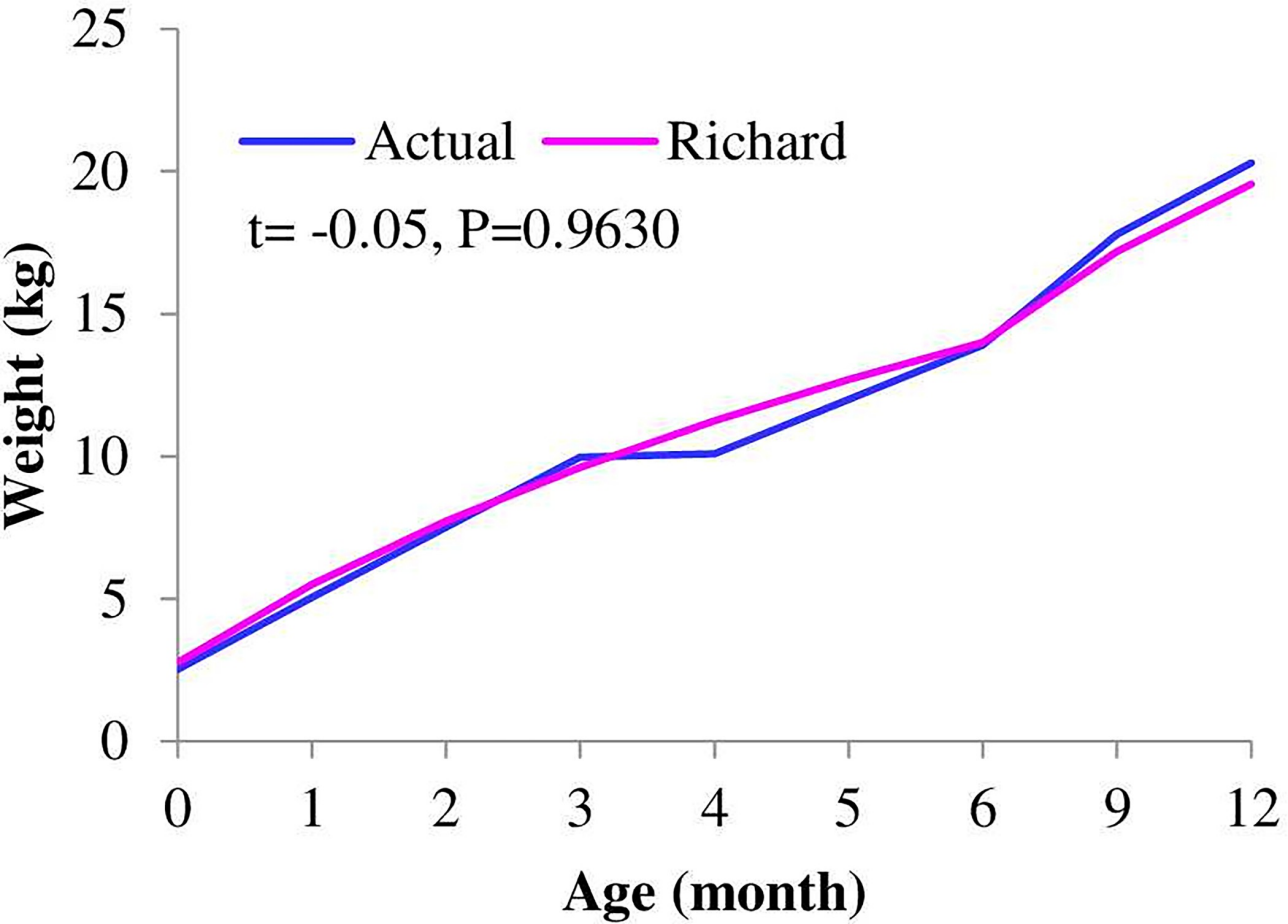
Actual and predicted bodyweights as a function of age obtained with a best-fitted model for F3 Boer x Central Highland goats.

**Fig 3:**
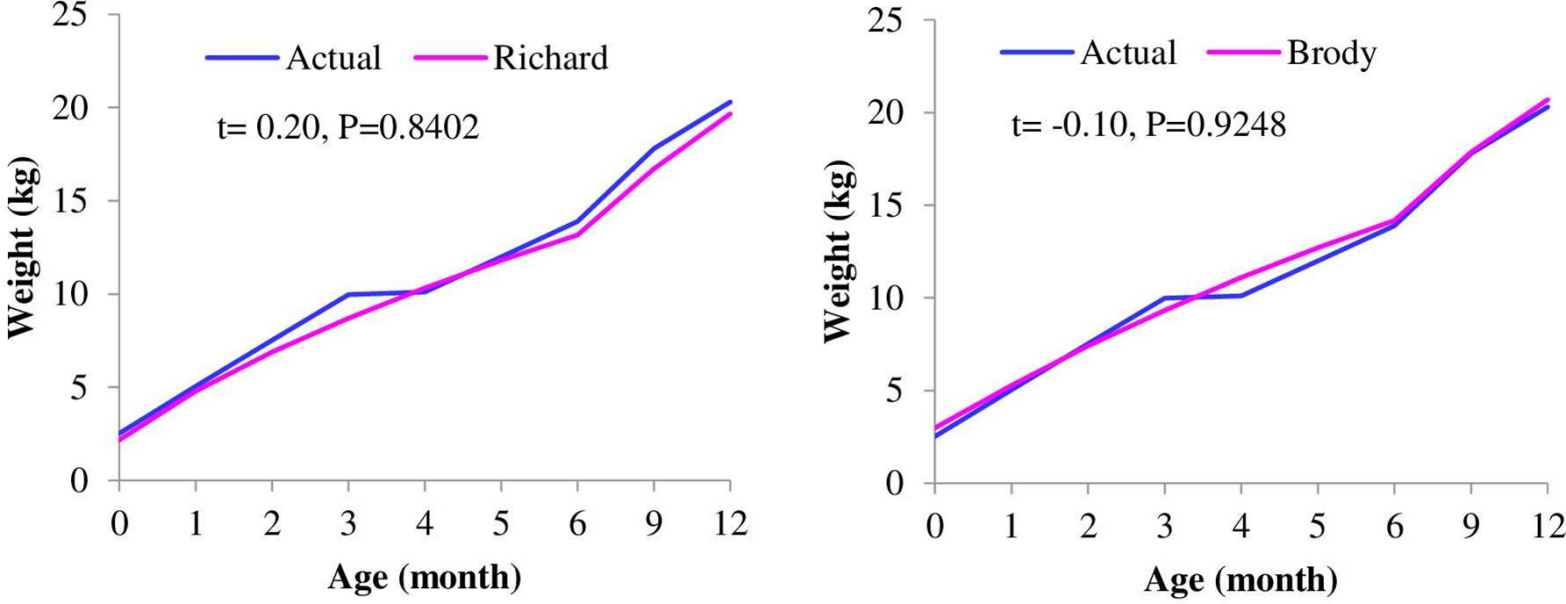
Actual and predicted body weights as a function of age obtained with a best-fitted model for female (left) and male (right) Boer x Central Highland goats.

Understanding the growth curve is important for culling and selection decisions. Predicted body weights (kg) as a function of age (months) obtained with best-fitted growth models for F1, F2, F3, male, and female kids are shown in Figs 1–3. The variation between the actual and predicted growth curves of crossbred goats increases with filial generation, although it was non-significant. The predicted body weight of females after five months was lower than the actual weight, while it was higher for males starting at three months. The growth was not similar in all age intervals. Growth increased at an increasing rate for up to six months, then increased at a decreasing rate for all genotypes and both sexes. This result is consistent with Trangerud et al. [41], who divide the growth curve into two phases, i.e., an early phase where the weight gain rate increases and a later phase where the weight gain rate decreases. This growth pattern suggests that keeping crossbred kids over six months of age induces more production costs per kg of meat; thus, keeping up to six months might be better because goats with faster growth can be slaughtered at a younger age, as they mature earlier than those with slower growth rates at the same initial weight. According to Berry et al. [42], reducing the number of days from birth to slaughter (i.e., fewer days on feed) may be one tactic to increase animal and herd level feed and environmental efficiency.

## Conclusion

Brody function was more versatile to fit the growth curve in Boer x Central Highland goats. The result based on the best-fitted growth function indicated that F1 goats had a higher mature weight than F2 and F3. However, the maturation rate tends to increase with the filial generation, which indicates the early maturation of F3 goats with low mature weight. Males had a higher mature weight than their female counterparts, although there was no difference in maturation rate among male and female goats. The growth curve may save time and cost, enabling the selection and culling of goats based on early growth parameters. Therefore, the Brody model can predict the mature body weight and maturation rate of Boer x Central Highland goats and be used to plan management and breeding strategies.

## Supporting information

S1 Data Body weight-age data. (XLSX)
